# Genome Sequence of *Bacillus alveayuensis* Strain 24KAM51, a Halotolerant Thermophile Isolated from a Hydrothermal Vent

**DOI:** 10.1128/genomeA.00982-15

**Published:** 2015-08-27

**Authors:** Sevasti Filippidou, Tina Wunderlin, Thomas Junier, Nicole Jeanneret, Shannon Johnson, Kim McMurry, Cheryl D. Gleasner, Chien-Chi Lo, Po-E Li, Momchilo Vuyisich, Patrick S. Chain, Pilar Junier

**Affiliations:** a Laboratory of Microbiology, Neuchâtel, Switzerland; b Vital-IT Group, Swiss Institute of Bioinformatics, Lausanne, Switzerland; c Los Alamos National Laboratory, Los Alamos, New Mexico, USA

## Abstract

*Bacillus alveayuensis* strain 24KAM51 was isolated from a marine hydrothermal vent in Milos, Greece. Its genome depicts interesting features of halotolerance and resistance to heavy metals.

## GENOME ANNOUNCEMENT

*Bacillus alveayuensis* is a thermophilic endospore-forming bacterium. The type strain MT1 was isolated from deep-sea sediment (1). However, to date, no genome is available for this species. Strain 24KAM51 was isolated from a hydrothermal vent on the coastal line of Alykes beach in Milos, Greece (36°42′353″ N, 24°28′197″ E, depth 1.5 m). It is able to grow at a temperature range of 30 to 80°C, with an optimum at 60°C. It can also tolerate acidic and alkaline conditions (pH growth range from 3 to 10), with an optimum at 7. Finally, it grows with up to 13% (wt/vol) NaCl. Based on 16S rRNA sequence identity, strain 24KAM51 is closely related to *B. alveayuensis* type strain TM1 (99% identity). A series of physiological tests also showed similarity to TM1. The genome of 24KAM51 was sequenced and annotated in order to contribute to a better understanding of the thermophilic and halotolerant lifestyle of extremophilic bacilli. To date, this is the only genome of *B. alveayuensis* publicly available*.*

Genomic DNA was extracted from an overnight culture using the QIAamp DNA minikit (Qiagen GmbH, Germany). For the draft genome of *B. alveayuensis* strain 24KAM51, an Illumina (2) short-insert (300 ± 70 bp) library was constructed and sequenced generating 43,458,058 reads, totaling 4.39 Mbp. The Illumina draft data were assembled with Velvet, version 1.2.08 (3). The estimated size of the genome is 6.7 Mbp, which provides 409× coverage of the genome. Genome annotation was performed using an Ergatis-based (4) workflow with minor manual curation and visualized with the Artemis genome browser and annotation tool (5). The G+C content is 38.1%. The complete genome sequence contained 6,597 genes, 10 rRNAs (5S, 16S, and 23S), 88 tRNAs, and 2 noncoding RNAs (ncRNAs) predicted. The genome of 24KAM51 was annotated, and its proteome revealed the presence of 198 genes related to the sporulation and germination pathways and 10 genes related to dipicolinic acid synthesis. Fifty-three proteins related to flagellar motility were found in the proteome of *B. alveayuensis* strain 24KAM51.

Many proteins are related to halotolerance in bacilli (6), some of which are present in multiple copies in the 24KAM51 proteome. More precisely, genes for Na^+^/H^+^ antiporters, proline/Na^+^ symporters, and glycine/betaine ABC transporters were found. Although isolated from a natural hot spring, its genome contains genes related to copper (copper-binding proteins and multicopper oxidase), manganese (manganese transporters and permeases), cadmium (cadmium transporter), zinc (zinc metalloproteases, proteases, and transporters), and arsenic (arsenic resistance protein, ArsB) resistance. Moreover, it possesses genes encoding the NarH and NarZ proteins (nitrate reduction), as well as DsrE (sulfur reduction).

### Nucleotide sequence accession numbers.

This whole-genome shotgun project has been deposited at DDBJ/EMBL/GenBank under the accession no. JYCE00000000. The version described here is JYCE00000000.1.
